# Use of Polycarbonate Waste as Aggregate in Recycled Gypsum Plasters

**DOI:** 10.3390/ma13143042

**Published:** 2020-07-08

**Authors:** Manuel Alejandro Pedreño-Rojas, Carmen Rodríguez-Liñán, Inês Flores-Colen, Jorge de Brito

**Affiliations:** 1 Departamento de Construcciones Arquitectónicas 1, Escuela Técnica Superior de Arquitectura, Universidad de Sevilla, Avenida Reina Mercedes, n_ 2, 41012 Sevilla, Spain; rlinan@us.es; 2 CERIS, Instituto Superior Técnico, Universidade de Lisboa, Av. Rovisco Pais, 1049-001 Lisboa, Portugal; ines.flores.colen@tecnico.ulisboa.pt (I.F.-C.); jb@civil.ist.utl.pt (J.d.B.)

**Keywords:** recycled gypsum, gypsum plaster, plastic waste, mechanical properties, SEM

## Abstract

The use of gypsum as an indoor coating material for buildings is very extensive. This means that huge amounts of gypsum waste are generated daily worldwide. Therefore, many researchers in the last years have been working on the generation of new gypsum-related materials and products that incorporate recycled gypsum waste as a replacement for the commercial one. On the other hand, trying to reduce the large amounts of plastic generated globally each year, several studies have used different types of plastic waste as aggregates for the development of new construction and building materials. However, up to now, no previous studies have been found in which any type of plastic waste has been used as an aggregate in a recycled gypsum matrix. This paper presents a study in which two different types of waste were mixed for the development of new gypsum plasters: unheated gypsum waste from industrial plasterboard production (GPW) and polycarbonate (PC) waste from rejected compact discs (CDs) and digital versatile discs (DVDs). In this sense, the mechanical and thermal performance of plasters was evaluated. Finally, in order to evaluate the changes in the microstructure of the composites, a scanning electron microscopy (SEM) analysis was conducted. The results showed a good performance of the new composites when both types of waste were combined in the mixes. New lightweight eco-efficient plasters, completely recycled, with enhanced flexural strength (by 14.8%), compressive strength (by 26.8%), and thermal conductivity (42.8% less), compared to the reference material, were achieved.

## 1. Introduction

The use of gypsum as an indoor coating material for buildings is very extensive. Its use in pastes, plastering mortars, or prefabricated elements (plasterboards, blocks) makes gypsum one of the most used materials on construction sites [1]. This means that huge amounts of gypsum waste are generated daily worldwide. According to Ahmed et al. [2], around 15 million tons of gypsum plasterboard waste end up in landfills each year worldwide.

Gypsum plaster for construction (CaSO_4_·0.5H_2_O, calcium sulfate hemihydrate/bassanite) is obtained by drying calcination of natural gypsum rock (CaSO_4_·2H_2_O, calcium sulfate dehydrate/gypsum) at 105 °C to 200 °C, as shown in Equation (1). After that, it must be mixed with water to be used, producing an exothermic reaction, described by Le Chatellier, in which the gypsum plaster rehydrates, becoming a hard material [3,4,5].

CaSO_4_ ·2H_2_O →(105 to 200 °C) → CaSO_4_·0.5H_2_O + 1.5H_2_O → CaSO_4_·2H_2_O + Heat (1)

As can be seen, the material chemical composition does not change, making it a completely recyclable product [6]. Therefore, many researchers in the last years have been working on the generation of new gypsum materials and products that incorporate recycled gypsum waste as a replacement of the original commercial one [7,8,9].

In this regard, Jimenez-Rivero and García Navarro [10,11,12,13] studied the environmental and economic cost of reusing gypsum plasterboard waste, concluding that more than 87% of that waste is sent to landfills in Europe. In a second stage, they developed new plasters and plasterboards using the gypsum waste as a partial substitute of a commercial one [14]. The results achieved by Camarini et al. were also very interesting. They analyzed the effects of recycling gypsum plasterboard waste from different points of view: influence of the heating time [15], thermal conductivity of the new composites [16], and the effects of citric acid as set retarder [17], among others.

The advantages of recycling that type of gypsum residue, in terms of waste reduction, energy consumption, and economics, have been analyzed by Fernandez-Casado [18]. Furthermore, the physicochemical and mechanical performance of gypsum plasters, with recycled gypsum waste, after multiple dehydration and hydration cycles, have been studied by Gerardo et al. [19], who did not observe an important loss in the mechanical properties of the new plasters after each cycle. In that sense, Erbs et al. [5] performed a similar study, in which they achieved 8.40 MPa of compressive strength of the plaster after three recycling cycles. This was well above the 2.00 MPa value established by the regulations as the minimum value for gypsum plasters.

As seen in Equation (1), recycled gypsums should be heated to be reused as a substitute of commercial gypsum. Therefore, Erbs et al. [8] evaluated various calcination temperatures (160 °C, 180 °C, and 200 °C) and times (1, 2, 4, 8, and 24 h) and concluded that the highest mechanical performance was achieved at 180 °C for 24 h. Later, fixing the calcination temperature (150 °C), Rosalí de Moraes Rossetto et al. [20] used different heating periods of the wastes (1, 2, 3, 4, 5, and 6 h) and found that when gypsum waste (from wall coverings, plasterboards and decorative ornaments) was used, the surface hardness and compressive strength of the plasters increased, while their workability worsened.

On the other hand, trying to reduce the large amounts of plastic generated each year worldwide, several studies have used different types of plastic waste as aggregates for the development of new construction and building materials [21,22,23,24]. Focusing on the investigations that used a gypsum matrix, one can differentiate the contributions that used foamed plastics (expanded polystyrene (EPS), extruded polystyrene (XPS), polyurethane (PUR), among others) from those that used more non-expanded ones (polyethylene terephthalate (PET), high-density polyethylene (HDPE), etc.).

The first group includes studies made by San-Antonio-González et al. [25,26], who used EPS and XPS as aggregates in the development of lightweight gypsum plasters with enhanced thermal properties. In this sense, González-Madariaga and Macia [27] also studied the influence of EPS waste in the generation of new gypsum panels for construction. Furthermore, PUR waste (up to 2 mm) was used as an aggregate in the gypsum matrix, obtaining composites and plasterboards with improved thermal behavior [28,29].

In 2006, Karaman et al. [30] obtained PET waste from recycled plastic bottles, using it as an aggregate in a gypsum matrix at weights of up to 20% of the added gypsum. Recently, Vidales-Barriguete et al. [31] recovered pellet waste (heterogeneous mixture of thermoset and thermoplastic polymers), using them as aggregates in gypsum plaster with enhanced mechanical properties. Finally, other researchers have developed flame-retardant bio-composites, trying to improve their behavior in the case of fire [32].

As demonstrated above, several works have studied, separately, the influence of plastic and gypsum waste in plasters. However, up to now, no previous study has been found in which any type of plastic waste has been used as an aggregate in a recycled gypsum matrix.

This paper presents the third step of research in which two different types of waste were mixed for the development of new gypsum plasters: unheated gypsum waste from industrial plasterboard production (GPW) and polycarbonate (PC) waste from rejected compact discs (CDs) and digital versatile discs (DVDs) [33]:

- In the first phase of the research, different percentages and sizes of polycarbonate waste were used as aggregates in a commercial gypsum matrix. New lightweight plasters with good mechanical performance and with improved thermal and environmental properties were obtained. The highest mechanical values were obtained in mixes with 10% (by weight of gypsum) of PC waste, while the best thermal and environmental performance was achieved for the plasters with 40% of plastic added [34];
- Second, a study in which gypsum waste was used as a partial substitute of commercial gypsum was conducted. Different heating temperatures and times were checked. It was concluded that it is possible to use unheated gypsum waste from plasterboards as a complete substitute of a traditional one, improving the environmental (77%), thermal (18.8%), and mechanical properties (17%) of the new plasters. However, the workability of the composites worsened [35,36].

Consequently, in this article, the effects of using both types of residues (GPW and PC), at different percentages, on the mechanical and thermal behavior of plasters was evaluated. Thus, the influence of a plastic aggregate (in this case, PC) in a recycled gypsum matrix was analyzed for the first time. Finally, in order to evaluate the changes in the microstructure of the composites, a SEM analysis was conducted.

## 2. Materials and Methods

### 2.1. Materials

**Figure 1:**
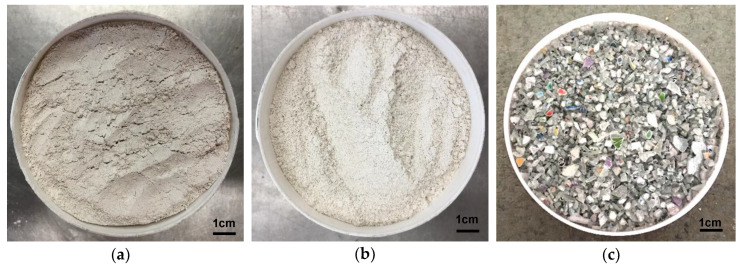
Materials used to develop the new plasters: (**a**) commercial gypsum; (**b**) unheated gypsum waste from plasterboard production; (**c**) polycarbonate waste.

For this study, the following materials were used for the development of the new gypsum plasters:

- *Commercial gypsum (CG):* traditional commercial gypsum for construction B1 [37], with controlled setting properties (Figure 1a);
- *Unheatedgypsum waste from plasterboard production (GPW):* acquired at a plasterboard manufacturing plant based in Sines (Portugal). The recycled gypsum came from the panels’ cutting process, using the pieces smaller than 1 mm (Figure 1b);
- *Polycarbonate waste (PC):* rejected CDs and DVDs obtained from all the recycling points located in the University of Seville (Spain). After that, the pieces were crushed, obtaining pieces smaller than 4 mm, as seen in Figure 1c.
- *Citric acid:* used as a set retarder in some composites to maintain the water/gypsum (w/g) ratio in all the plasters [17,35].

The chemical composition of both types of gypsum used was obtained using the x-ray diffraction (XRD) technique. For this purpose, a XPERT-PRO PANALYTICAL diffractometer from the Laboratory of Mineralogy, Petrology, and Geochemistry of Instituto Superior Técnico (LAMPGIST) was used.

**Figure 2:**
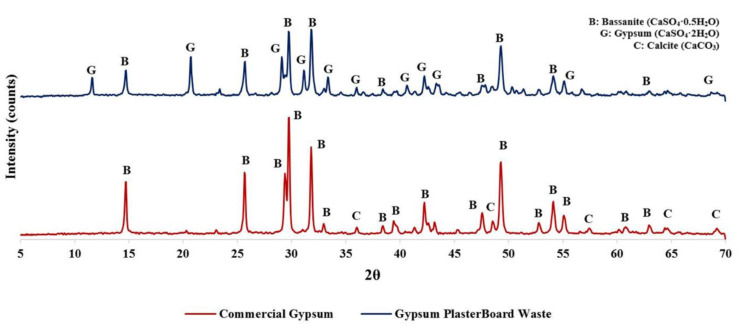
X-ray diffraction (XRD) of both types of gypsum waste used.

Figure 2 presents the diffractogram of both materials where it can be noticed that the commercial gypsum (from Escayescos production plant, located in Alcaudete, Spain) was mostly 100% hemihydrated gypsum (bassanite, CaSO_4_·0.5H_2_O) with a small amount of calcite (CaCO_3_), while GWP is a mixture of dihydrate particles (gypsum, CaSO_4_·2H_2_O) and bassanite.

### 2.2. Plaster Preparation

Different gypsum plasters [37,38] were produced by mixing different contents of polycarbonate waste, commercial gypsum, and recycled gypsum. The composition of all the mixes under study is presented in Table 1.

The w/g ratio of the plasters was obtained using the flow-table test, following the procedure defined in UNE-EN 13279-2 [38]. As noted in previous studies, the use of gypsum waste worsened the workability of the new plasters, requiring more water to make the mixes [35]. Therefore, and in order to maintain the w/g ratio in all the plasters, citric acid was used as a set retarder of the composites. According to previous studies, it was used as 1‰ per weight of the recycled gypsum used in the mixes [19].

Following these proportions, three 40 × 40 × 160 mm^3^ prismatic samples per mix were produced for the physical and mechanical characterization of the new plasters [38]. In addition, 70 mm round samples, 20 mm thick, were prepared to test the thermal conductivity of the composites.

### 2.3. Test Methods

**Figure 3:**
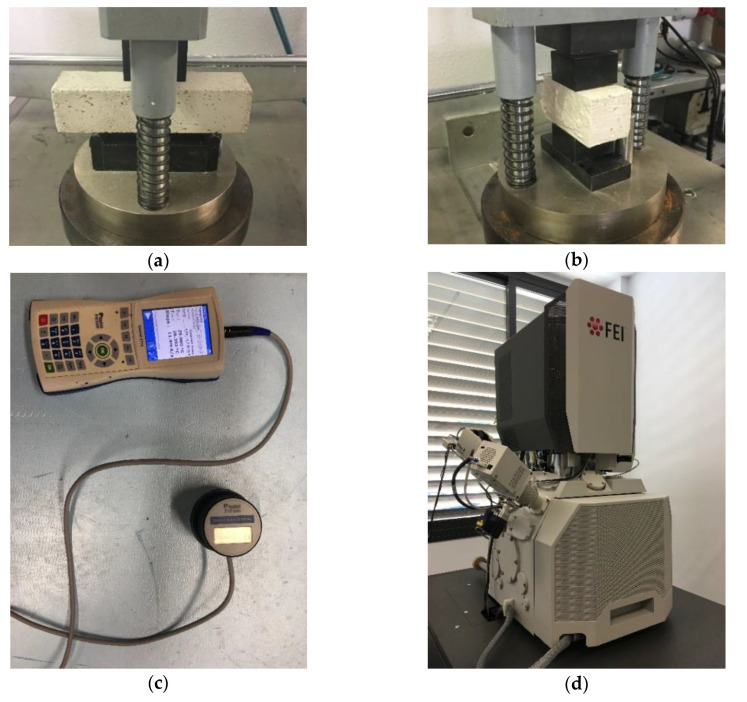
Test methods: (**a**) Flexural strength; (**b**) Compressive strength; (**c**) Thermal conductivity; (**d**) scanning electron microscopy (SEM).

Following the procedure defined in UNE-EN 13279-2, the specimens were placed at 24 °C and 50 ± 1% of relative humidity for seven days. Then, they were put in an oven at 40 ± 2 °C to reach constant mass [38]. After that, the new plasters were characterized, analyzing the following properties:

- *Dry bulk density:* according to the method described in UNE-EN 13279-2 [38], density is defined as the ratio between the dry mass of the sample and its volume;
- *Flexural strength:* the three-point bending test was used to measure the flexural strength of the new plasters, as defined by the regulation [38] (Figure 3a);
- *Compressive strength:* after the three-point bending test pieces were broken, the resulting six halves were subjected to a progressive centered load test [38] until the compressive strength of the composites was determined (Figure 3b);
- *Thermal conductivity:* this was obtained by using the ISOMET-2114 device (located in Instituto Superior Técnico, Lisbon, Portugal) (Figure 3c), in accordance with the procedure described in ASTM D5930-09 [39];
- *Scanning electron microscopy (SEM):* a FEI TENEO microscope (located in Universidad de Sevilla, Seville, Spain) was used to carry out the SEM analysis of the new plasters (Figure 3d).

## 3. Results and Discussion

### 3.1. Dry Bulk Density

**Figure 4:**
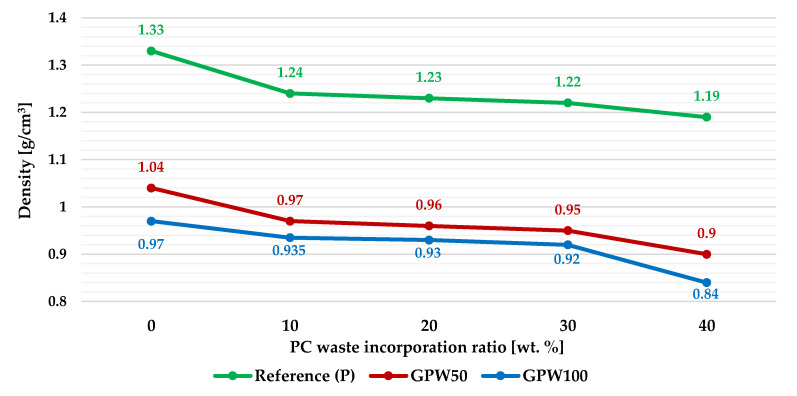
Dry bulk density of the developed gypsum plasters.

Figure 4 presents the dry bulk density results of the new gypsum mixes. It is concluded that when the percentage of PC waste added to the plasters increased, the density of the material decreased. Therefore, the highest decrease was obtained for the GPW100 P40 mix, in which the density of the plaster was 36.8% lower than the one obtained for the reference material. In addition, the differences between the density values achieved for the GPW100 and GPW50 plasters, with the same percentage of plastic waste added, was not very significant compared to the one obtained for the reference series (without recycled gypsum).

### 3.2. Flexural Strength

**Figure 5:**
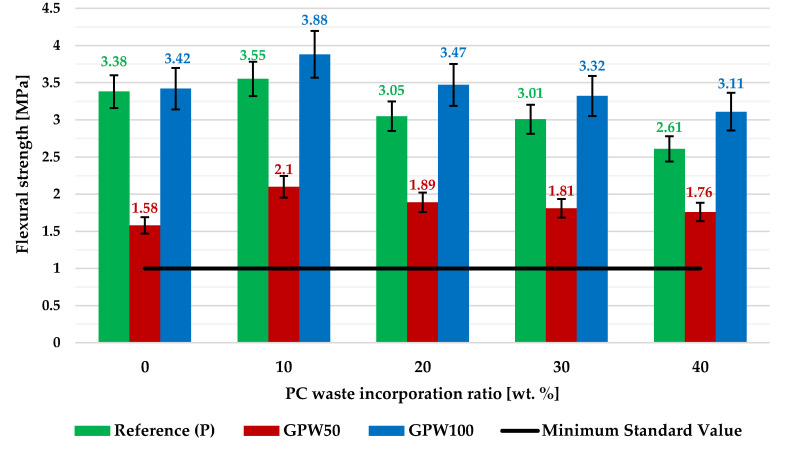
Flexural strength of the developed gypsum plasters with error bars.

Figure 5 presents the flexural strength results with error bars (standard deviation). It shows that increasing the amount of polycarbonate waste added to the plasters resulted in a drop in flexural strength of the new plasters. In all of the mixes, and for the same waste content, plasters with complete substitution of the commercial gypsum with a recycled one (GPW100 series) showed the best mechanical behavior. On the other hand, GPW50 mixes presented the worst capacity. According to the results, the biggest rise in the strength performance of the plasters compared to the material without the plastic aggregate was achieved in samples with 10% PC waste, with the biggest growth (32.9%) found in the GPW50-P10 composite. Furthermore, all mixes with 50% of gypsum waste (GPW50 series) that contained a PC aggregate showed an improvement in their flexural strength capacity relative to the one without plastic waste (GWP50), which achieved the lowest value (1.58 MPa). Finally, all the newly developed composites achieved the minimum 1 MPa standard requirement for gypsum plasters, thus being fit to be used in any construction work [37].

### 3.3. Compressive Strength

**Figure 6:**
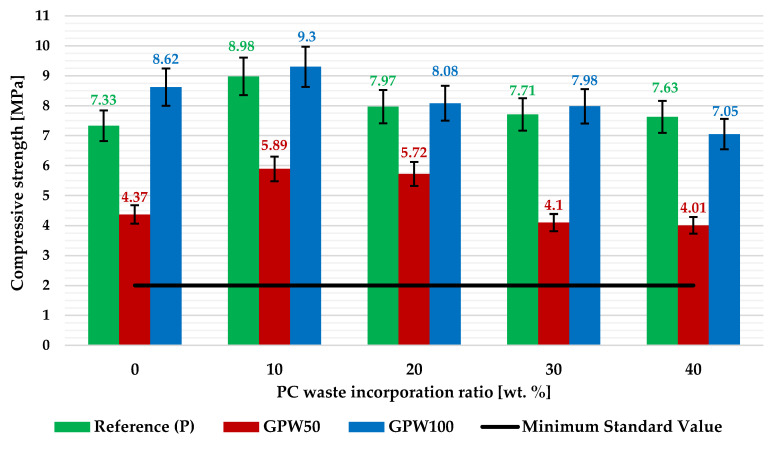
Compressive strength values for the developed gypsum plasters with error bars.

The compressive strength results of the gypsum plasters are presented in Figure 6 with error bars (standard deviation). In general terms, the same behavior as in the bending test was detected. A slight improvement of the resistance values was obtained, in all cases, when 10 wt% PC was used as an aggregate in the mixes. Once again, the GPW100-P10 mix was the one with the best performance (9.30 MPa), with an improvement of 26.9% relative to the reference sample. On the other hand, the worst compressive strength result was obtained for the GPW50-P40 composite at 4.01 MPa. Furthermore, it is important to highlight again that, for all the developed plasters, the minimum 2 MPa value required by the standard for the compressive strength of gypsum plasters [37] was noticeably exceeded. Thus, all of these are ready for use as a construction material with a wide number of applications.

### 3.4. Thermal Conductivity

**Figure 7:**
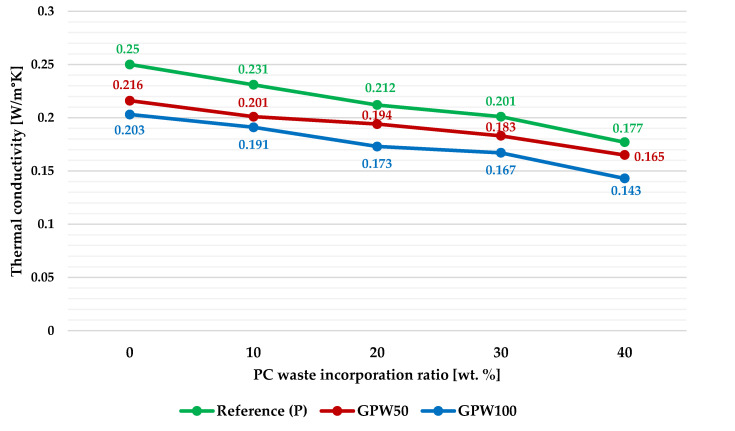
Thermal conductivity of the developed gypsum plasters.

According to the results obtained in this test (Figure 7), it can be said that a slight drop in thermal conductivity of the new gypsum composites was observed as the amount of both types of wastes added (GPW and PC) increased. Thus, the best values were achieved for GPW100-P40 (0.143 W/m°K), in which an enhancement of 42.8% was obtained relative to the reference plaster (0.250 W/m°K).

### 3.5. Scanning Electron Microscopy (SEM) Analysis

**Figure 8:**
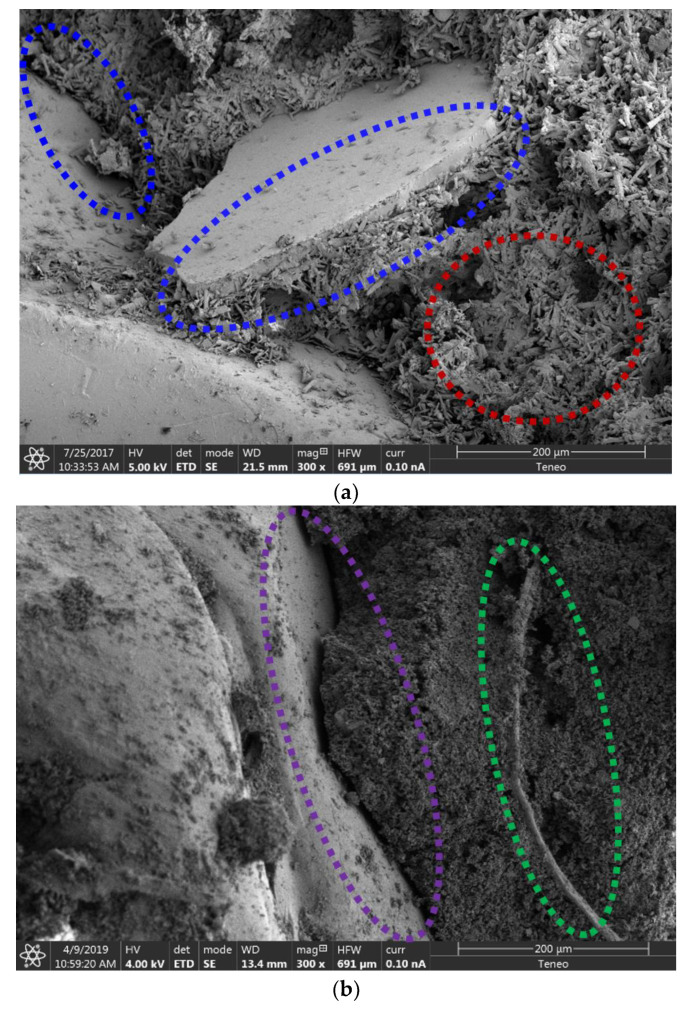

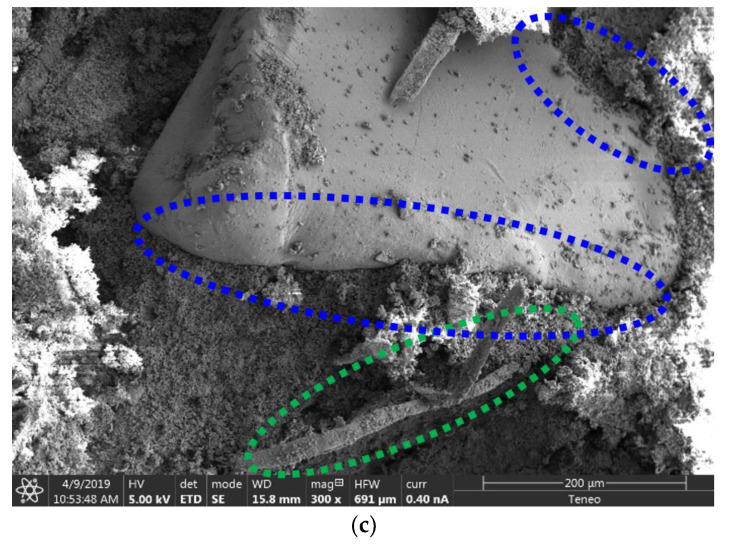
SEM images of the gypsum plasters (300×): (**a**) P20; (**b**) GPW50-P20; (**c**) GPW100-P20. Highlights: crystalline gypsum structure (red); good adherence between the PC and the gypsum matrix (blue); glass fiber (green) and bad adherence between the matrix and the plastic aggregate (purple).

To clarify some of the values achieved in the mechanical performance tests, a SEM analysis was carried out for samples with 20% PC waste out of the entire series (P20, GPW50-P20, and GPW100-P20). Figure 8 shows some of the images taken in the analysis. First of all, it must be said that an important change in the gypsum matrix microstructure was detected when gypsum waste was used as a substitute for a commercial one and citric acid as the set retarder. The crystalline structure seen in the P20 images was completely different from the one obtained for the other plasters. In addition, the adherence on the interface between the gypsum matrix and the PC particles seemed to be excellent in the P20 and GPW100-P20 samples. However, a deterioration of the adherence was observed in the GPW50-P20 samples. This can justify the results obtained in the mechanical tests, where a drop in strength occurred in the GPW50 series. It could be said that the union of both types of gypsum residues generated an internal structure that worsened the mechanical capabilities of the new plasters [35,36], also decreasing the adhesion with the added PC. Finally, some glass fibers were seen in plasters with GPW, improving the bending properties of the plasters as it presented a good adherence to the gypsum matrix.

## 4. Conclusions

In this paper, new gypsum plasters were produced using gypsum waste as the total or partial replacement of commercial gypsum and polycarbonate waste as the aggregate in the mixes. According to the results obtained during the experimental program, the following conclusions can be drawn:

- For all new composites, the increase in content of both types of residues added was linked to a decrease in dry bulk density of the plasters. In addition, that drop was more relevant in composites in which the amount of recycled gypsum used was higher. Consequently, the highest decrease was obtained for the GPW100 P40 mix, in which the density of the plaster was 36.8% lower than the one obtained for the reference plaster;
- According to the mechanical performance tests, it was noticed that some of the developed plasters exceeded the values of the reference composite. For both tests, flexural and compressive strength, the highest increase was obtained for the GPW100 P10 plaster (reaching 3.88 MPa and 9.30 MPa, respectively), so these plasters can have different applications; and
- The thermal conductivity test showed that all of the newly developed plasters presented a significant improvement in this property relative to the value of the reference plaster, and the GPW100 P40 composite was the one with the lowest coefficient (0.143 W/m°K).

Summing up, new lightweight eco-efficient plasters, completely recycled, with enhanced flexural strength (by up to 14.8%), compressive strength (by up to 26.8%), and thermal conductivity (up to 42.8% less), relative to the reference material, were developed. This means that the newly developed materials contribute to significantly reduce the amounts of this waste that ends up in landfills, contributing to a circular economy, while simultaneously achieving a notable enhancement in their physical and mechanical properties.

## Figures and Tables

**Table 1:** Composition of all the gypsum mixes under study.

Sample		Commercial Gypsum [g]	Gypsum Waste [g]	Water [g] or [mL]	w/g Ratio	Citric Acid [g]	Polycarbonate Waste [g]
**Reference**		1000	-	550	0.55	-	-
**P10**		1000	-	550	0.55	-	100
**P20**		1000	-	550	0.55	-	200
**P30**		1000	-	550	0.55	-	300
**P40**		1000	-	550	0.55	-	400
**GPW50**		500	500	550	0.55	0.5	-
**GPW50**	**P10**	500	500	550	0.55	0.5	100
	**P20**	500	500	550	0.55	0.5	200
	**P30**	500	500	550	0.55	0.5	300
	**P40**	500	500	550	0.55	0.5	400
**GPW100**		-	1000	550	0.55	1	-
**GPW100**	**P10**	-	1000	550	0.55	1	100
	**P20**	-	1000	550	0.55	1	200
	**P30**	-	1000	550	0.55	1	300
	**P40**	-	1000	550	0.55	1	400

## References

[1] De Brito J., Flores-Colen I. (2015). Gypsum plasters. Materials for Construction and Civil Engineering.

[2] Ahmed A., Ugai K., Kamei T. (2011). Investigation of recycled gypsum in conjunction with waste plastic trays for ground improvement. Constr. Build. Mater..

[3] Fořt J., Černý R. (2018). Carbon footprint analysis of calcined gypsum production in the Czech Republic. J. Clean. Prod..

[4] Gartner E.M. (2009). Cohesion and expansion in polycrystalline solids formed by hydration reactions—The case of gypsum plasters. Cem. Concr. Res..

[5] Erbs A., Nagalli A., de Carvalho K.Q., Mymrin V., Passig F.H., Mazer W. (2018). Properties of recycled gypsum from gypsum plasterboards and commercial gypsum throughout recycling cycles. J. Clean. Prod..

[6] GtoG Project. http://gypsumtogypsum.org/.

[7] Begliardo H., Sanchez M., Cecilia Panigatti M., Garrappa S. (2013). Reuse of recovered construction gyp-sum plaster: A study based on aptitude requirements of argentine and Chilean standards. Rev. Constr..

[8] Erbs A., Nagalli A., Mymrine V., Carvalho K.Q. (2015). Determination of physical and mechanical properties of recycled gypsum from the plasterboard sheets. Cerâmica.

[9] Papailiopoulou N., Grigoropoulou H., Founti M. (2017). Energy analysis of the effects of high-level rein-corporation of post-consumer recycled gypsum in plasterboard manufacturing. Waste Biomass Valorization.

[10] Jiménez-Rivero A., García-Navarro J. (2016). Indicators to measure the management performance of end-of-life gypsum: From deconstruction to production of recycled gypsum. Waste Biomass Valorization.

[11] Jiménez-Rivero A., Sathre R., García-Navarro J. (2016). Life cycle energy and material flow implications of gypsum plasterboard recycling in the European Union. Resour. Conserv. Recycl..

[12] Jiménez-Rivero A., García-Navarro J. (2017). Exploring factors influencing post-consumer gypsum recycling and landfilling in the European Union. Resour. Conserv. Recycl..

[13] Jiménez-Rivero A., García-Navarro J. (2017). Best practices for the management of end-of-life gypsum in a circular economy. J. Clean. Prod..

[14] Jiménez-Rivero A., García-Navarro J. (2017). Characterization of quality recycled gypsum and plasterboard with maximized recycled content. Mater. Constr..

[15] Camarini G., Pinheiro S.M., Tannous K. (2013). Thermal analysis of recycled gypsum from construction and demolition waste. Appl. Mech. Mater..

[16] Camarini G., dos Santos Lima K.D., Pinheiro S.M. (2016). Investigation on gypsum plaster waste recycling: An eco-friendly material. Green Mater..

[17] Camarini G., Pinto M.C.C., de Moura A.G., Manzo N.R. (2016). Effect of citric acid on properties of recycled gypsum plaster to building components. Constr. Build. Mater..

[18] Fernández Casado S. Reciclaje interno de los residuos en las fábricas. Reutilización del yeso reciclado para la fabricación de placas de yeso laminado. Proceedings of the Congreso Nacional del Medio Ambiente.

[19] Geraldo R.H., Pinheiro S.M., Silva J.S., Andrade H.M., Dweck J., Gonçalves J.P., Camarini G. (2017). Gypsum plaster waste recycling: A potential environmental and industrial solution. J. Clean. Prod..

[20] Rosalí de Moraes Rossetto J., Santos Correia L., Henrique Geraldo R., Camarini G. (2016). Gypsum plaster waste recycling: Analysis of calcination time. Key Eng. Mater..

[21] Saikia N., de Brito J. (2012). Use of plastic waste as aggregate in cement mortar and concrete preparation: A review. Constr. Build. Mater..

[22] Saikia N., de Brito J. (2014). Mechanical properties and abrasion behaviour of concrete containing shredded PET bottle waste as a partial substitution of natural aggregate. Constr. Build. Mater..

[23] Siddique R., Khatib J., Kaur I. (2008). Use of recycled plastic in concrete: A review. Waste Manag..

[24] Rubio-de Hita P., Pérez-Gálvez F., Morales-Conde M.J., Pedreño-Rojas M.A. (2018). Reuse of plastic waste of mixed polypropylene as aggregate in mortars for the manufacture of pieces for restoring jack arch floors with timber beams. J. Clean. Prod..

[25] San-Antonio-González A., Del Río Merino M., Viñas Arrebola C., Villoria-Sáez P. (2015). Lightweight material made with gypsum and EPS waste with enhanced mechanical strength. J. Mater. Civ. Eng..

[26] San-Antonio-González A., Del Río Merino M., Viñas Arrebola C., Villoria-Sáez P. (2015). Lightweight material made with gypsum and extruded polystyrene waste with enhanced thermal behaviour. Constr. Build. Mater..

[27] González Madariaga F.J., Macia J.L. (2008). EPS (expanded polystyrene) recycled bends mixed with plaster or stucco, some applications in building industry. Inf. Constr..

[28] Gutiérrez-González S., Gadea J., Rodríguez A., Junco C., Calderón V. (2012). Lightweight plaster materials with enhanced thermal properties made with polyurethane foam wastes. Constr. Build. Mater..

[29] Alameda L., Calderón V., Junco C., Rodríguez A., Gadea J., Gutiérrez-González S. (2016). Characterization of gypsum plasterboard with polyurethane foam waste reinforced with polypropylene fibers. Mater. Constr..

[30] Karaman S., Sahin S., Gunal H., Orung I. (2006). Stabilization of waste pet bottles with Gypsum. J. Appl. Sci..

[31] Vidales Barriguete A., del Río Merino M., Atanes Sánchez E., Piña Ramírez C., Viñas Arrebola C. (2018). Analysis of the feasibility of the use of CDW as a low-environmental-impact aggregate in conglomerates. Constr. Build. Mater..

[32] Anannya F.R., Mahmud M.A. (2019). Developments in Flame-Retardant Bio-composite Material Production. Adv. Civ. Eng. Mater..

[33] Antonakou E.V., Kalogiannis K.G., Stephanidis S.D., Triantafyllidis K.S., Lappas A.A., Achilias D.S. (2014). Pyrolysis and catalytic pyrolysis as a recycling method of waste CDs originating from polycarbonate and HIPS. Waste Manag..

[34] Pedreño-Rojas M.A., Morales-Conde M.J., Pérez-Gálvez F., Rubio-de-Hita P. (2019). Influence of polycarbonate waste on gypsum composites: Mechanical and environmental study. J. Clean. Prod..

[35] Pedreño-Rojas M.A., Flores-Colen I., De Brito J., Rodríguez-Liñán C. (2019). Influence of the heating process on the use of gypsum wastes in plasters: Mechanical, thermal and environmental analysis. J. Clean. Prod..

[36] Pedreño-Rojas M.A., De Brito J., Flores-Colen I., Pereira M.F.C., Rubio-de-Hita P. (2020). Influence of gypsum wastes on the workability of plasters: Heating process and microstructural analysis. J. Build. Eng..

[37] UNE-EN 13279-1 (2006). Gypsum Binders and Gypsum Plasters—Part 1: Definitions and Requirements.

[38] UNE-EN 13279-2 (2006). Gypsum Binders and Gypsum Plasters—Part 2: Test Methods.

[39] ASTM D5930-09 (2009). Thermal Conductivity of Plastics by Means of a Transient Line-Source Technique.

